# Supplementary material for: OpenMM 7: Rapid development of high performance algorithms for molecular dynamics
Source: PLoS Comput Biol. 2017 Jul 26;13(7):e1005659. doi: 10.1371/journal.pcbi.1005659 (PMC5549999; doi:10.1371/journal.pcbi.1005659)
Supplement: S1 Source Code — (ZIP) [file pcbi.1005659.s002.zip › OpenMM-7.0.1-Source/docs/api-c++/generated/OpenMM.CMMotionRemover.html]

CMMotionRemover — OpenMM @OPENMM\_MAJOR\_VERSION@.@OPENMM\_MINOR\_VERSION@ documentation


# CMMotionRemover¶

*class* `OpenMM::``CMMotionRemover`¶
:   This class prevents the center of mass of a `System` from drifting. At each time step, it calculates the center of mass momentum, then adjusts the individual particle velocities to make it zero.

    Methods

    |  |  |
    | --- | --- |
    | `CMMotionRemover` | Create a `CMMotionRemover`. |
    | `getFrequency` | Get the frequency (in time steps) at which center of mass motion should be removed |
    | `setFrequency` | Set the frequency (in time steps) at which center of mass motion should be removed |
    | `usesPeriodicBoundaryConditions` | Returns whether or not this force makes use of periodic boundary conditions. |

    `CMMotionRemover`(int *frequency* = 1)¶
    :   Create a `CMMotionRemover`.

    int `getFrequency`() *const*¶
    :   Get the frequency (in time steps) at which center of mass motion should be removed

    void `setFrequency`(int *freq*)¶
    :   Set the frequency (in time steps) at which center of mass motion should be removed

    bool `usesPeriodicBoundaryConditions`() *const*¶
    :   Returns whether or not this force makes use of periodic boundary conditions.

        |  |  |
        | --- | --- |
        | Returns: | true if force uses PBC and false otherwise |

High performance molecular simulation on GPUs


### Navigation

- Core Objects
- Forces
- Integrators
- Extras

©2015, Stanford University and the Authors.
|
Powered by Sphinx 1.3.1
& Alabaster 0.7.7
|
Page source
